# Discovering Periodic Patterns in Historical News

**DOI:** 10.1371/journal.pone.0165736

**Published:** 2016-11-08

**Authors:** Fabon Dzogang, Thomas Lansdall-Welfare, FindMyPast Newspaper Team, Nello Cristianini

**Affiliations:** 1 Intelligent Systems Laboratory, University of Bristol, Bristol, BS8 1UB, United Kingdom; 2 FindMyPast Newspaper Archive Limited, Gateway House, Luna Place, Technology Park, Dundee, DD2 1TP, Scotland, United Kingdom; Columbia University, UNITED STATES

## Abstract

We address the problem of observing periodic changes in the behaviour of a large population, by analysing the daily contents of newspapers published in the United States and United Kingdom from 1836 to 1922. This is done by analysing the daily time series of the relative frequency of the 25K most frequent words for each country, resulting in the study of 50K time series for 31,755 days. Behaviours that are found to be strongly periodic include seasonal activities, such as hunting and harvesting. A strong connection with natural cycles is found, with a pronounced presence of fruits, vegetables, flowers and game. Periodicities dictated by religious or civil calendars are also detected and show a different wave-form than those provoked by weather. States that can be revealed include the presence of infectious disease, with clear annual peaks for fever, pneumonia and diarrhoea. Overall, 2% of the words are found to be strongly periodic, and the period most frequently found is 365 days. Comparisons between UK and US, and between modern and historical news, reveal how the fundamental cycles of life are shaped by the seasons, but also how this effect has been reduced in modern times.

## Introduction

Observing changes in some state or behaviour of a large population is a task tackled by different research communities using various disparate means: the start of an epidemic may be detected by analysing data from hospital admissions [1], a shift in public opinion may be discovered by telephone polls and surveys [2–4], and an increase in criminal behaviour can be detected by analysing police records. Sometimes even indirect information can be useful to this effect: changes in society can be detected by observing sales of a given product over time [5], by comparing electoral results [6] and stock markets [7], or by analysing web searches [8,9] and social media content [10–13]. For a few quantities, such as for example the price of wheat or daily temperatures, high quality records have been kept for a long time, and have proven valuable for historians and social scientists. Changes, continuities and fluctuations in those quantities can be highly meaningful if interpreted in the correct context. However, for most aspects of social behaviour we do not have a direct way of recording them as they happen: why would anyone directly measure how much time people spent in church or at a pub, over many decades? Over very short time periods this has been attempted by the Mass Observation project [14] but this approach cannot be applied over the long periods that are typical of major social changes.

This is where a new source of data can be invaluable: the contents of regional daily newspapers provide an indirect, but nevertheless informative, record of everyday life for long periods of time.

With the automatic digitisation of large quantities of historical newspapers and books, it has recently become possible to analyse historical data which indirectly reflect the conditions of a population at a given time and place.

Previous studies conducted on the contents of historical books [15] and newspapers [16] have already revealed continuities, changes and events, including linguistic evolution and cultural shifts, through the impact they left in the text.

An important class of behaviours and phenomena are those that occur periodically: for studies about climate and economy, these may include seasonal cycles, and their accurate observation can help to understand the mechanisms behind them. In the case of collective behaviour within a society, these may include all sorts of activities that are repeated at regular intervals, and could lead to an understanding of the mechanisms that may cause large amounts of people to engage in the same activities at the same time, and at regular intervals. For example, recent studies on the content of online activities have revealed clear cycles in certain behaviours, such as smoking cessation and mental health information seeking [17,18], and have used those observations to propose possible mechanisms behind them. A further study [19] analysed four daily time series of sentiment extracted from Twitter in the UK, representing the amount of negative affect, anxiety, anger and sadness expressed in the collective discourse. This revealed a strong seasonal structure to mood, particularly showing winter peaks for these negative emotions. It also reported a similar seasonality in Wikipedia searches that are related to mental health.

In this study we address the problem of detecting periodic activities at a whole population level by analysing time series of words frequencies extracted from historical newspapers spanning a long period of time. We are interested in following the relative frequency of each word on each day over a period of many decades, to detect those words that show a significant periodic pattern. The final goal is not just to detect those periodic words, but to understand the social reality behind them, and to shed light on the underlying social synchronisation mechanism responsible for their periodic expression.

The technical challenge is both of a statistical and computational nature, and it is so general that its solution can be directly applied to many other cases where massively multivariate time series are analysed. This is the case, for example, in the analysis of stock market data, sensor networks data and financial transactions.

We propose a general method to discover statistically significant periodic patterns in these massively multivariate time series, and we use it to demonstrate how historical newspapers can be used to understand cycles in collective behaviour. In particular, we discovered that a large number of activities were taking place in a seasonal fashion during the 87 years under investigation, and were mostly synchronised with the environment. These included leisure, or agriculture or cultural activities. We further demonstrated how these cyclic behaviours have now changed, with activities being more synchronised by cultural phenomena like the football season.

In order to detect periodicity in the textual time series we make use of the theory of Fourier transforms [20]. Any time series can be decomposed into a linear combination of simpler sine and cosine components that oscillate at each harmonic frequency. The Fourier coefficients associated with each frequency component form the Fourier Spectrum of the time series (sometimes known as its frequency domain representation), and provide information about the proportion of variance explained by a sinusoidal waveform, along with the peak time in the period of oscillation.

**Fig 1:**
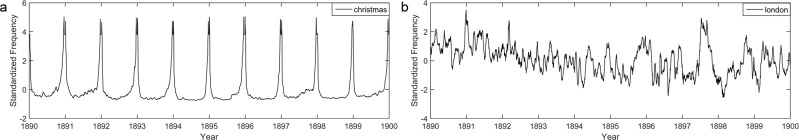
An example of two different word time series as extracted from historical newspapers in the United Kingdom. (a) a time series of a strongly periodic word (Christmas), and (b) a time series which is not periodic (London). Both time series in this example are shortened to the 10 years between 1890 and 1900 for illustrative purposes.

Taking this approach we automatically discover periodic patterns in time series of daily word frequencies extracted from millions of pages of digitised historical newspapers in two distinct corpora, one originating from the United Kingdom and the other from the United States, both covering the same period in history. Specifically, the United Kingdom corpus consists of 28.5 billion words from 121 different news outlets, while the United States corpus contains 45.6 billion words from 1,635 news outlets. For each of the corpora we analyse the daily time series of news content in the 87 year interval between 1836 and 1922. We focus on the 25,000 most common words, resulting in the analysis of 50,000 time series, each consisting of 31,755 daily readings, and we concentrate on cycles between 30 days and 5 years in order to compare findings across countries and time. An example showing two of these time series is shown in Fig 1.

## Data Description

This study is based on daily time series extracted from news content, as well as some publically available weather time series. We have made the frequency time series used in this study openly available [21].

### Historical news content

We used two distinct historical newspaper corpora, one originating from newspapers in the United States of America, the other from the United Kingdom. Additionally, for comparison with the historical news, we gathered a modern day news corpus from the web, along with historical daily temperature, precipitation and photoperiod time series.

For the United States time series, we processed 8.9 million pages of historical newspaper coverage made available through the Chronicling America Project. The corpus covers sets of newspaper pages which have been selected to best represent different states’ regional history, geographical coverage and the events that happened during an 87-year period of interest between 1836 and 1922. Raw OCR text is made available from the digitized pages, following technical specifications designed by the Library of Congress. The corpus of documents from the Chronicling America Project used in this study contains all data in batches released up to and including 2015-07-22 from the Bulk Data JSON feed at http://chroniclingamerica.loc.gov/ocr.json. Further information about the Chronicling American project can be found at http://chroniclingamerica.loc.gov/about/.

For the United Kingdom time series, we processed 33.9 million articles of historical newspaper coverage made available through the British Newspaper Archive. The corpus covers a selection of newspaper issues which were selected to best represent different regions of the United Kingdom, taking into account the completeness of the run of each issue, the number of years each issue covers and the quality of the OCR for an issue. While a greater range of years was available for the British corpus, we limited the time interval to the same 87 years between 1836 and 1922 as the US corpus to allow the time series from the corpora to be directly comparable. Raw OCR text was processed from the digitized articles, following technical specifications agreed between the British Newspaper Archive and the British Library. Further information about the British Newspaper Archive project can be found at http://www.britishnewspaperarchive.co.uk/help/about.

For the modern day UK time series, we processed 2.7 million English-language news articles from 1,231 news outlets that have a web presence and are published in the United Kingdom, covering the six-year period between 1st January 2010 and 31st December 2015. News articles were gathered from the Really Simple Syndication (RSS) feed for the front page of the news outlets, representing the top stories on their website, an analogous concept to the front page of a newspaper. Collection of the articles was performed by our modular system for news media analysis [22]. This process involved selecting feeds for news media outlets by crawling a number of seed webpages, such as lists of newspapers on Wikipedia [23], and manually adding them to the system. Following this, each news article which appeared in any of the news feeds during the period was retrieved, extracting the main text of the news article by separating it from non-content textual information, such as comments or navigation, using an adaptation of the text-to-tag ratio [24] which extracts the largest set of text with a common parent within the HTML structure.

### Weather observations

Weather information for the United Kingdom corresponds with daily observations from the Met Office Hadley Observation Centre [25,26]. Historical observations were available in the period between 1st January 1878 and 30th December 1922 for the temperature and precipitation level. Modern measurements were available in the period between 1st January 2010 and 31st December 2015, matching our modern news time series. The data are representative of a triangular area of the United Kingdom enclosed by Lancashire, London, and Bristol.

Weather information for the United States was obtained from the National Centers for Environmental Observations of the US National Oceanic and Atmospheric Administration [27]. Historical weather observations were taken in the period between 1st January 1893 and 30th December 1922 for all stations in the United States available in that time. Daily estimates for the temperature and precipitation level across the continent were aggregated and used to describe weather in that location.

Photoperiod (day-length) was computed [28] using the same time interval used for weather observations, in two representative locations, London in the United Kingdom and New York City in the United States.

## Methodology

### Overview

We analysed time series extracted from two large-scale historical corpora consisting respectively of 28.5 and 45.6 billion words covering the years from 1836 to 1922 (31,755 days) collected from 121 UK and 1,635 US newspapers. Our analysis can be broken down into two key steps: one aimed at the estimation of the daily relative frequency of each word which is detailed in the word time series generation section, the other aimed at the detection of significant periodicities in the resulting time series, described in the Fourier analysis and significance testing sections. The first step starts with the raw counts of a word in a day, and involves normalisation, smoothing, de-trending and standardisation of the time series. The second one applies the fast Fourier Transform to each time series, and then performs statistical hypothesis testing. The key challenge here is to account for the massively multiple statistical testing.

### Word time series generation

Time series for each word were generated for each corpus independently in the following way. Text from each corpus was first tokenized using the Word Break rules from the Unicode Text Segmentation algorithm, following the specification in the Unicode Standard Annex #29 (http://unicode.org/reports/tr29/). Each token was further processed to remove possessives (trailing ‘s at the end of words), lowercased and stemmed using the Porter stemmer algorithm [29], as is standard in text mining applications. The number of times a word occurred on a given date was computed across all articles or pages, using the date assigned to the article or page, resulting in a raw count for the number of times a word was mentioned per day, as well as the total count for the entire corpus. The 29th February was removed from each leap year to keep the time series aligned. The sum for the total number of words used per day was also computed, and we refer to this as the daily volume. Days which contained no data, due to the corpus containing no data for that specific date, had the raw count and daily volume interpolated using linear interpolation to fill in the gaps. We focused on the 25,000 most commonly occurring words in each corpus, before removing the 260 words from the US corpus and 567 words from the UK corpus with a gap of longer than nine years. A 15-day centred moving average was applied to both the raw counts and the daily volume in order to improve the estimation of a word’s frequency. The raw counts for each word were then divided by the daily volume on each corresponding date to give the relative frequency times series of each word. The main concern in this step was to ensure that we have enough statistics to estimate the relative frequency of a word, where many of the words will be rather rare due to Zipf’s law. This was one of the key reasons why we limited our analysis to the most commonly occurring words, and introduce the 15-day moving average.

### Pre-processing of the time series

As is standard in spectral analysis [30], we further de-trended the relative frequency series, performed by applying a 10-year centred moving average to the relative frequency series to obtain a trend time series. The original relative frequency series was de-trended by subtracting the corresponding trend time series for the word. The de-trended time series of each word was standardized before clipping values lying outside three standard deviations, to reduce the effect of rare extreme events on the result. A final standardisation step is performed for convenience.

The 15-day centred moving average that is applied defines the smallest resolution that we can discover for the period of a word, while the 10-year de-trending step defines the largest resolution for discovered periodicities. Therefore, with these parameters, we certainly have removed any periodic signal lower than 15 days and higher than 10 years. To stay in a safe interval of frequencies, and avoid boundary effects, we double the lower threshold and half the higher one, only considering cycles over 30 days or below 5 years.

### Fourier analysis

We performed a standard fast Fourier transformation [20] on the time series of each word [21] (24,433 words in the UK corpus, 24,740 in the US corpus), obtaining for each word the spectrum of the Fourier frequencies deemed resolvable by the Nyquist sampling Theorem [31]. We focused on periods between 30 days and 5 years (double the size of the smoothing window of 15 days, and half the size of the de-trending applied) as explained above. The spectrum was normalised so that each component could be interpreted as the proportion of variance explained in a time series by the corresponding frequency. At this point we have a Fourier spectrum for each of the words in the two corpora, and the next stage is to perform a multiple statistical test as described below. We should note that periodicity of a time series is a matter of degree: while we test all the words, our analysis then focuses only on those words that have a sufficiently large component in the spectrum, so that a large part of their variance can be explained by a single component.

### Statistical significance testing

We divide the statistical testing procedure into two steps: computing p-values for a single word, and accounting for multiple testing. For the first step, we use the magnitude of the largest component in the Fourier spectrum as the test statistic, and a null hypothesis that the data was sampled i.i.d. from a distribution with the same properties as the given data (white noise). The p-value is the probability that the spectrum of a time series generated by the null hypothesis has a peak equal or larger than the one observed.

One way to generate a time series according to the null hypothesis is to randomly permute it, generating a white-noise time series, whose spectrum is known to be uniform. We also assume that the magnitude of the largest peak, over many random permutations, will follow an Extreme Value Distribution (EVD), an assumption that we verified numerically by chi-squared test. In order to fit an EVD to the given data, we performed 1,000 random permutations for each time series, computing the test statistic each time, and then fitting the EVD by maximum likelihood. This was then used to calculate the probability of the observed value of test statistic under the null model. For each word we find a p-value that is less than 4.65e^-10^.

For the second step, we note that this procedure has been repeated for each of the 25,000 words in each corpus, therefore leading to a massive number of multiple tests. We use the Bonferroni correction to account for this, and find that the probability of a non-periodic word being added to our list of periodic words is less than 1%.

In order to double check the above results, a smaller set of words was tested even further, using a different null hypothesis and a brute force calculation of the p-value. This was done by permuting the raw word count time series before following the same processing steps as in the original study, instead of permuting the relative frequency time series. This gives rise to a null hypothesis closer to the concept of background noise, rather than white noise. The brute force approach consisted in sampling 2.5 million random permutations and assessing whether the test statistic, the largest component, in any of our random Fourier spectrums is equally as large or larger than for the original time series. Again, this p-value is then corrected using the Bonferroni correction to account for the massive number of multiple tests we are performing. We obtained identical results, once more showing that we could not explain any of our findings with the (alternative) null hypothesis. Hence, using both approaches, we found that all words that we identified as periodic are significant when maintaining the probability of at least one false rejection at the significance level of 1% after the number of multiple tests performed.

### Boundary effects in the frequency domain

While the above procedure was designed just to identify the strongest components of each word, provided they are in the interval between 30 days and 5 years, we should note for the sake of being exhaustive that if we expanded the analysis to consider any frequency, we would see that a number of unrelated words display a (very weak) peak at 3175.5 days, which is exactly 1/10th of the full time series, and which seems to be due to an artefact. This is probably a systematic effect of our pre-processing steps and finite sample sizes. We inspected those time series, and found no periodic pattern in the time domain. This period is outside the interval of frequencies under investigation, and was not included in any successive analysis.

## Results and Discussion

Fourier analysis of the 50,000 time series extracted from our two corpora reveals some striking properties. First of all, while a large proportion of words exhibit a weak periodicity, we discover that there is a subset of about 2% of the words which are strongly periodic, which we define as having more than 20% of their variation explained in terms of one single component. In an even smaller set of words, over 90% of the variation can be explained by using the 5 largest components.

More specifically, at least 5% of the variance can be explained by a single component for 21% of the words in the UK and 42% in the US, a single component explains at least 10% of the variance for 5% of the words in UK and 10% in the US while 20% of the variance can be explained by a single component for 1.6% of words in the UK and 2.1% in the US. Remarkably 16 words in the UK (27 in the US) can account for more than 50% of their variation using only a single component. As a reference, the time series of daily temperature for the same historical period in the UK can explain 84% of its variance with a single component corresponding to a yearly period, while the variation in the daily precipitation level is only explained 1%. In other words, certain words, and by inference certain human activities, are at least as periodic as the weather.

**Fig 2:**
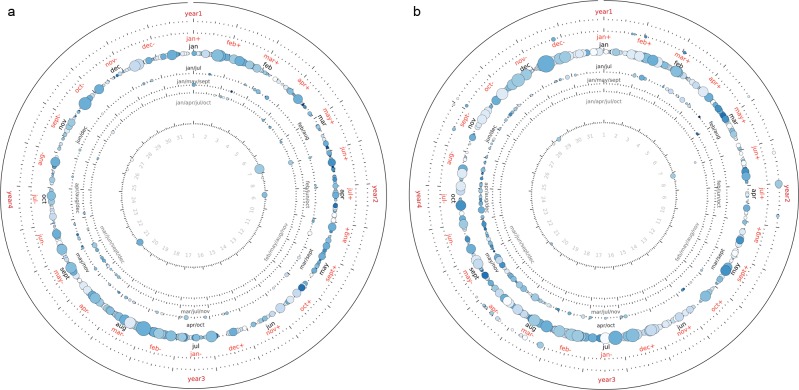
**Periodicities of words in (a) the UK corpus and (b) the US corpus, with at least 20% of their variance explained by a single Fourier component.** Each circle represents a single word, with its position around each figure indicating the peak time of usage, the radius from the centre indicating the period between 30 days (inner most) and four years (outer most), while the size of the individual circles, along with their colour indicates the variance explained, with larger, darker circles explaining more of their variance with a single Fourier component. This shows the general methodology we developed for the detection of periodic words is capable of finding any periodicity between 30 days and 5 years, with a rigorous statistical test. Some words have indeed a 2 years cycle (US political elections) and some others have a shorter cycle (fashion related words). This makes more remarkable the finding that most of the periodic words have a 12 month cycle. Similarly, there seems to be a non-uniform distribution in the phases, with most words peaking in the summer months.

Of those periodic words, an important finding is that 15% in the US and 20% in the UK are best explained by a 365-day period, although other secondary periods can be detected in those words, while a small number are best explained by a 30-day, six-month, two-year and even four-year period (Fig 2). This indicates that there is a small set of cycles behind many human activities. Furthermore, the phase (time of peaking) of these periodic components is not distributed uniformly across the year. Most yearly words tend to peak either in the summer or in the winter. While certain phenomena and activities clearly peak in the spring and autumn, the majority of words tend to peak in July or December. Further analysis of the peaking time of words is detailed in the section on comparisons with temperature, photoperiod and precipitation.

### Strongly periodic words

**Fig 3:**
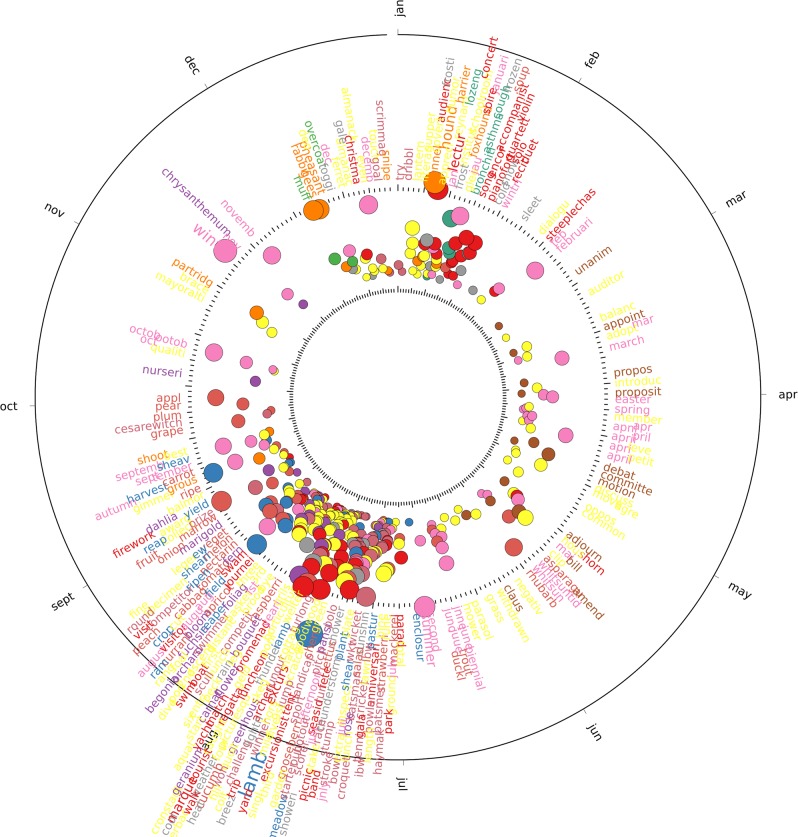
Strongly periodic words in the UK corpus with a seasonal component are shown categorised into one of 12 topical categories. Each circle denotes a word in the corpus, with the labels shown around the outside. Label font size and circle size indicate the variance explained by the first component. Position around the figure indicates the phase of the period, corresponding to the day of the year where the word is most present. Words are grouped by colour indicating their category.

**Fig 4:**
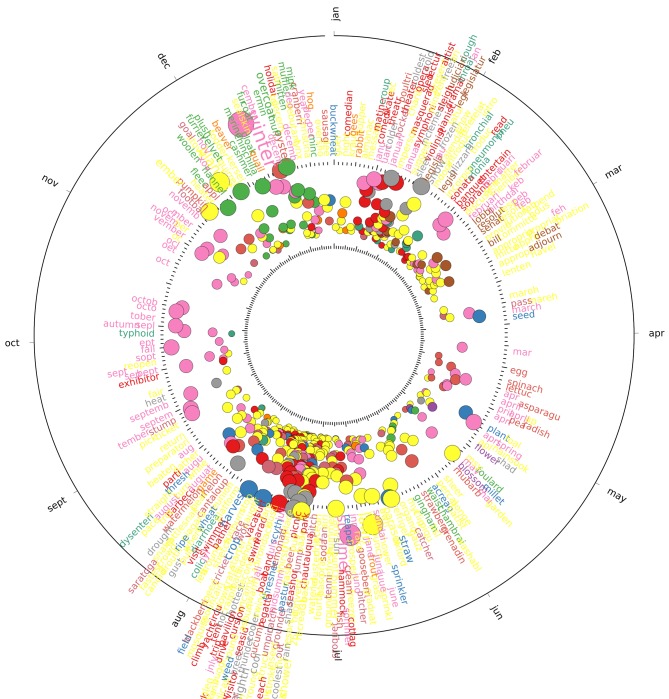
Strongly periodic words in the US corpus with a seasonal component are shown categorised into one of 12 topical categories. Each circle denotes a word in the corpus, with the labels shown around the outside. Label font size and circle size indicate the variance explained by the first component. Position around the figure indicates the phase of the period, corresponding to the day of the year where the word is most present. Words are grouped by colour indicating their category.

The set of strongly periodic words in both UK and US corpora include the names of the months, the four seasons and religious festivities such as Christmas and Easter (all of which have a phase corresponding to the expected time of year in a 365-day period). Besides these obvious words which provide a useful sanity check, we detect a strong, yearly periodic component in words related to leisure activities, agriculture, hunting, food, and weather, as well as the periodic seasonal return of certain infectious diseases. Figs 3 and 4 show the words from each corpus that are strongly periodic with a 365-day period, plotted by their phase and percentage of variance explained, manually coloured by their relation to the categories we identified, with all remaining words grouped into the ‘other‘ category. A full list of all words found in each category mentioned here can be found in S1 and S2 Tables.

Leisure activities in the UK and US display strongly periodic behaviour, with words relating to outdoor leisure activities such as fete, picnic, excursion and trip appearing most frequently in the summer, while during the winter indoor activities become commonplace, including opera, pantomime, theatre and soiree. This contrasts with outdoor work activities which take place as required, and are mostly associated with agriculture, such as harvest and the other related words of crop, reaper and thresh mostly occurring every year around July and August, or animal care such as shear and shearling also peaking every July and August.

The activity of hunting game and other animals is also strongly periodic within the UK corpus, indicating its importance as an outdoor winter activity with words relating to quarry generally appearing towards the middle of their open season [32], with pheasant and geese peaking in December, grouse in September and partridge in November, while references to dogs typically used for hunting such as foxhounds and harriers appear in January. In the US there are fewer strongly periodic words relating to hunting and quarry, although those that do appear, such as rabbit, geese, quail and beaver, peak during the winter months.

Food related words in the UK exhibit a strong connection to the availability of different fruits and vegetables during the seasons, with the majority of these words concentrating their phase between July and September when the fruit and vegetables would typically be harvested [33] including cucumber, tomato, onion, strawberry, plum, and cherry. A similar pattern can be seen in the US corpus, although some of the food related words can be found peaking earlier in the year, such as strawberry in May, and asparagus, radish and lettuce in April. Other plants such as flowers also follow their corresponding seasonal patterns [34] in the corpora, including geraniums and roses peaking in July, carnations and marigolds in August and chrysanthemums appearing later in the autumn.

One remarkably periodic word that does not seem to be directly related to any religious or agricultural calendar is the word lecture, which peaks in early January in both the UK and US corpora, and where 55% of the variance can be explained by a single component in the UK, and 31% in the US. We have not found it to relate to any one specific event, such as the Royal Institution’s traditional Christmas lecture, but rather to an increased number of lectures taking place all around the country through the period of the New Year. Upon close reading, these were found to be as diverse as lectures on tuberculosis, radium and x-rays or foreign travel delivered in Esperanto, and fit with the general idea of a Victorian era romance with festive science [35, 36].

### Testing disease words for seasonality

Some disease and symptom related words were found to be strongly periodic with a 365-day period, including cough, bronchitis and asthma all peaking in January in the UK; while in the US cough, pneumonia, croup and bronchial peak in January and early February; colic, diarrhoea and dysentery peak in August; and typhoid peaks during October.

We additionally tested the periodicity of other disease-related words, besides those strongly periodic words directly discovered by our method. The words we tested were chosen because they refer to illnesses that are claimed to be seasonal in epidemiological papers [37, 38], and we wished to ascertain if they do have a cyclic behaviour within our corpora (even if only a small part of their variance can be explained by a single component), and if their peak season reflects the general opinion of epidemiologists.

Of the 10 diseases and symptoms which are believed to be seasonal in the articles we consulted [37], we discovered that seven of those were seasonal in at least one of the corpora, one (diphtheria) was not, and two (rotavirus and gonorrhoea) could not be tested because they did not occur frequently enough. Cholera was found to have a seasonal component that explained between 9% (UK) and 15% (US) of its variance, peaking around August and September; measles explained between 4% (UK) and 17% (US) of its variance with a seasonal component with a peak in late March to early April; while between 6% (UK) and 15% (US) of the variance of the word fever can be explained by a seasonal component peaking in either November for the UK or September in the US. Pox, malaria and infection were all found to have no seasonal component in the UK corpus, but had 9%, 15% and 4% respectively of their variance explained by a seasonal component in the US corpus, with malaria peaking in August, infection peaking in September and pox in February. Influenza was found not to follow a seasonal pattern in the US corpus, while in the UK 11% of its variance was explained by a seasonal pattern peaking in February.

Additionally, we wished to test the periodicity of the word death, to see if this followed a seasonal pattern in our corpora as has been observed in various studies [39–41] that often indicate winter months as the peak time for death by disease, and warmer months for violent death. We found that death can explain 4% of its variance using a seasonal component in the UK, with a peak in January matching the phase reported in other literature [42]. In the US however, death is best explained (3%) by a six month component, with peaks in early February and early August, which appears to coincide with the phase of the respiratory and gastrointestinal diseases we found to be strongly periodic. This is a weak signal however, so further work needs to be done to separate the various causes of death.

While a certain amount of variance in disease-related words can be explained by a seasonal cycle, this is not particularly strong, suggesting that seasonality is only one of the factors influencing epidemics or other causes of death. However, the time of peaking we found is compatible with the time we would expect for measles, chickenpox (late winter and early spring), and fever and influenza (cold months).

We also should note that there is a methodological difference when performing statistical testing for words which are discovered from the data to be strongly periodic, and those which we shortlisted *a priori* as is the case for these disease related words. This difference refers to the way we account for multiple testing, since the p-value is affected by the size of the list.

### Comparisons across datasets

Comparing the time series from the two historical corpora with each other, we can find differences in the behaviours and activities performed in the past, allowing us to compare across different geographical regions. Additionally, using a corpus of modern news gathered from the web, we investigate words which continue to be periodic now, and those which ceased to be periodic.

#### Comparing historical news between the UK and US

When comparing UK with US historical news, we can see important differences in the words which are strongly periodic. Many of these differences can be accounted for by linguistics, climate or customs. For example, the word seashore is strongly periodic (35%) within the US, with a peak in mid-July, while this word is not present within the UK. However, we do find the word seaside is strongly periodic (34%) for the UK corpus, with a peak in mid-July within two days of the US peak for seashore.

Climate differences between the UK and US are evidenced by the strongly periodic mention of particular fruits including watermelon and cantaloupe exclusively being periodic in the US, or nectarines exclusively being periodic in the UK. Looking specifically at the words for the seasons, we see that whilst summer peaks in both countries between 25^th^ June (UK) and 28^th^ June (US), the other seasons all peak one month earlier in the UK, suggesting that the UK summer, from the peak of summer on 25^th^ June to the peak of autumn on 10^th^ September, was considered a month shorter than in the US, lasting from 28^th^ June to the peak of autumn on 4^th^ October.

Differences in customs between the two countries can be found in larger sets of related words (topics) which are strongly periodic and peak together around the same time of year, but only exist in one of the countries. One of the most obvious of these is the appearance of many baseball related words in the US (umpire, pitcher, base etc.) peaking around July, whereas the UK shows a peak of cricket related words at the same time (wicket, stump, runout etc.). Other topics which appear more periodically every year in only one of the countries include gardening related words in the UK (flower names, horticulture, etc.) and fashion related words in the US (stylish, tailor, millinery etc.). A full list of periodic words which appear in only one of the corpora can be found in S1 and S2 Datasets.

#### Comparing past with present

We also compared the historical UK news with modern day UK news gathered from 1,231 UK news outlets on the web covering a period of six years between 1st January 2010 and 31st December 2015, to see if anything has changed in the periodic structure of news content, which might signal a change in culture or behaviour over the past century. For example, we discovered that the word lecture mentioned earlier no longer appears as periodic in the modern news, whilst it was found to be one of the most strongly periodic words in the past, suggesting the cultural phenomenon of festive science is a lost tradition, whereas pantomimes, which were periodic in the past (20%), remain periodic (23%) in modern news. Other lost traditions that emerge strongly from the dataset include gooseberries, a traditional British summer fruit which was periodic before (46%), but is no longer found in the modern corpus and whose disappearance from the shops has been lamented in the media [43]; hunting activities and their associated quarry, with many of the words related to hunting no longer being periodic, including both the quarry (pheasant, partridge, snipe etc.) and the dogs used for hunting (such as foxhounds and harriers); and the tradition of passing the time in the colder months with music concerts (as evidenced by concert, audience, accompanist etc.).

Other traditions and customs in Britain appear to have persisted over the last century, including the national pastimes of gardening, as evidenced by many related words such as horticulture, garden and greenhouse remaining strongly periodic in the modern news from the UK, and talking about the weather, indicated by words such as sunshine, snow and thunderstorm also remaining strongly periodic today.

We can also look at the opposite comparison, investigating words that mark our seasons now, but did not do so in the past. These include skier and sled in the winter and barbecue and Spain in the summer. Additionally, we see a large number of words relating to imported traditions from other cultures in the modern news such as Halloween, Oktoberfest, Ibiza, grotto, Santa and reindeer. Indeed, we can see that whilst pumpkin was never periodic in the historical UK news, it was found to be periodic in the US news, with 32% of its variance explained by a seasonal component with a peak in November, and has now been incorporated into UK culture, with 20% of its variance explained by a seasonal component and a peak in late October in the modern UK news.

It seems that while the news now still shows a strong seasonality, the “pacemaker” has changed: periodic words related to hunting and agriculture have mostly disappeared, while mention of the weather has been joined with a sports season. At the same time a new set of imported activities has joined the list of events that mark the passing of seasons. A full list of all words which have disappeared from, or been introduced to the modern UK news can be found in S3 and S4 Datasets.

#### Comparisons with temperature, precipitation, and photoperiod

As we found that the changing of the seasons appears to be marked by new events and activities in the modern news, we wished to check if historically the weather might have driven the changes of activity and behaviours throughout the year. Essentially, are the seasonal patterns found in word frequencies a reflection of the weather or of the time of year? To answer this question, we first measured the correlation between all periodic words in the UK and US historical news with the time series of daily temperature and photoperiod (day-length) in the respective geographical regions, along with a historical records of daily precipitation in the UK.

Temperature and photoperiod time series are both very close to sinusoidal, with a period of 365 days, the first one peaking in late July, and the second peaking on June 21st. The difference between their phases is approximately a month. The variance in temperature can be explained for 73% in the UK, 93% in the US by a single Fourier component, while the photoperiod is a sine wave by construction. Daily precipitation is much less periodic, although when smoothed over many contiguous days it does show a clear seasonal pattern, peaking in early November.

In the historical UK news, we find that the words with the highest correlation with temperature are cucumber, lawn, and cricket, while the words with the lowest correlation are snow, recital and concert. Similarly, correlation with the photoperiod is highest for the words summer, lamb and cricket, which is perhaps unsurprising given the high correlation between temperature and photoperiod. The highest correlation with precipitation are all words related to raining, including rain, flood, and torrential.

**Fig 5:**
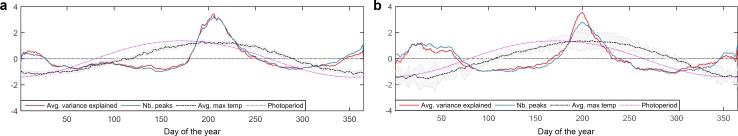
**Variance explained averaged over all seasonally periodic words and total number of seasonally periodic words peaking per day in (a) the UK corpus and (b) the US corpus, compared with the maximum daily temperature and photoperiod in each geographical region respectively.** Each series is standardized while the two former series were first smoothed using a 15-day centred moving average. The grey area represents the 99% confidence interval for the Temperature series. We can see that the average variance explained per day and the number of seasonal words peaking per day correlates more closely with the temperature, rather than the photoperiod, with most words peaking at the warmest and coldest time of the year. This suggests that activities are driven by temperature, rather than by day-length.

However, most words that have a 365-day period peak either in the summer or in the winter. It is interesting to observe that the periods when most words peak coincide with the warmest and the coldest time of the year, suggesting that activities are driven by temperature, rather than by day-length (Fig 5).

## Non Sinusoidal Waveforms

It should not be surprising that a single Fourier component is not sufficient to explain all the variance in any of our words. Indeed, this should be expected only for a perfectly sinusoidal and infinite time series. Since we have a finite time series, and since many of the periodic words do not follow a simple sinusoid, we do have various Fourier components activated. Besides creating the risk of spurious peaks in the spectrum, and complicating our estimate of phase, this effect also downplays the importance of words that are periodic but not sinusoidal. It is important to note that any abrupt change in the time series will introduce some spurious Fourier components.

**Fig 6:**
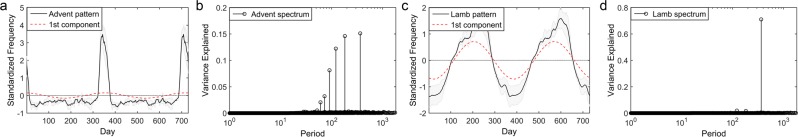
Examples of sinusoidal and complex waveforms in the time domain, and their Fourier spectrums from the UK corpus. (a) shows the most complex periodic waveform (advent) in the time domain, while (b) shows the corresponding Fourier spectrum where we can see several components are activated. (c) shows the most sinusoidal periodic waveform (lamb) in the time domain, with (d) showing the corresponding Fourier spectrum where we can see that only one component is strongly activated, explaining nearly 80% of the variance in the time domain.

We are also interested in detecting words that are clearly periodic, but have a more complex waveform. Examples of the most sinusoidal (lamb) and least sinusoidal (advent) time series are shown, along with their Fourier spectrum in Fig 6. These can be seen as words whose behaviour needs to be explained by more than one Fourier component. We do this by computing how much variance of their time series can be explained by combining the five largest Fourier components. We find that in this way we can explain over 75% of the variation in words such as Christmas and Harvest, and around 90% for most month names.

In order to isolate the words that have a complex waveform from the sinusoidal words, we compare the variance explained by the first component with the variance explained by the next four components. We find that most calendar events such as the names of each month, and festivities like Advent, Christmas and Easter, have non-sinusoidal waveforms. Christmas in particular has a nearly-triangular wave, due to the build-up of anticipation in the weeks and months leading up to it, followed by an abrupt drop. On the other hand, words like lamb or cricket, do seem to follow a much more sinusoidal pattern, being very much related to season or weather.

### Cycles of different period

Expanding our analysis from focusing on yearly periodicities, we can also look at words which follow a longer or shorter cycle than one year. The processing steps that are used in our data analysis mean that it may be difficult to reliably detect periods shorter than about a month and longer than a few years. This is due to an initial 15 day moving average performed to the word time series, and a ten year de-trending step used to remove long-term changes over the 87 year period we study (see Methods for further details). Nevertheless, our analysis did find strongly periodic words with super-annual periods of two and four years, as well as strongly periodic words repeating every six months.

The two and four year periodicities occur within the US corpus, and relate to the political cycle of presidential elections and the midterm elections that happen every four years, with a two year offset in the phase between them. The word presidential (37%) is the most strongly periodic of the words discovered with a four year period, peaking in December every fourth year, and is supported by many related words with the same period and similar phase including electorate (16%), vice (12%), nation (8%) and contest (7%). Similarly, every two years we find evidence of another political cycle with words including chairman (20%), primary (16%), vote (15%), support (11%) and congress (10%). We found no evidence of a similar super-annual political cycle within the UK corpus at a five year period as we might expect. We believe this is due to snap elections being called, or general elections following failure to pass motions of confidence causing the UK general elections to not follow a fixed period.

At the six month period, we see evidence of fashion seasons within the US, with words such as millinery (36%), stylish (34%), latest (34%), trim (33%), and many more semantically related words occurring around the same time, with peaks around April/May and October/November each year, suggesting there is a Spring-Summer fashion season and an Autumn-Winter fashion season in effect. This is accompanied by the words reduced (26%) and clearance (24%) also being explained by a six month component with a phase that peaks in January and July each year, approximately half way through each fashion season.

## Conclusions

The Fourier analysis of the relative frequency of 50K words over a period of 31,755 days reveals that a significant fraction of these words follows a strongly cyclic behaviour. The most common period is 365 days, and the most common phases are those peaking in the summer and in the winter. The activities underlying the periodic words are often related to the seasons: we see names of fruits and flowers, outdoor activities, agricultural tasks. We also see signs of seasonal disease, and the strong signals related to calendar-driven activities, such as Christmas celebrations. These general properties can be observed in the time series extracted both from the UK and from the US corpus, and to some extent also in those extracted from modern news. Interestingly, however, many of the underlying drivers have changed in modern times, with current periodicities being more informed by events not related to the natural world.

It is tempting to suggest that the pace of human activities is today less strongly set by natural cycles: the passage of time in historical newspapers was marked by produce like gooseberries and rhubarb, and activities like hunting, harvesting and playing cricket, or events like lectures and concerts. It now appears to rely less on these natural cycles, instead introducing new periodic elements to set their pace, including Halloween, Santa Claus, Football Championships and summer travel.

Not only does all this provide important material for social scientists and historians to investigate, it also shows that collective behaviour in societies does indeed display a periodic pattern, and that the analysis of newspaper time series can uncover at least a part of it.

It is also important to observe that the method we have developed for the statistical handling of such a large multiple testing problem is entirely general, and therefore could be used in many other domains where massively multivariate time series need to be analysed. This may include the detection of cycles in credit card transactions, media consumption, stock markets.

We expect them to be useful to analyse economic indicators, social media and sensor data. Among the scientific disciplines that can benefit from our proposed periodic models, are fields including economics, anthropology, social sciences, the emerging subfields of culturomics and cliodynamics [44], as well as financial markets analysis to name a few.

We believe that while the automation of text analysis can never replace the role of human scholars, it can play an important role in the digital humanities by detecting macroscopic trends that might otherwise go unnoticed, bringing them to the attention of the historian or the social scientist.

## Supporting Information

S1 Dataset Periodic words unique to the UK. Periodic words that appear in the UK historical corpus, but are not present periodically in the US historical corpus.
(CSV)

S2 Dataset Periodic words unique to the US. Periodic words that appear in the US historical corpus, but are not present periodically in the UK historical corpus.
(CSV)

S3 Dataset Historically periodic words in the UK. Seasonally periodic words that appear in the UK historical corpus, but are not present periodically in the modern UK corpus.
(CSV)

S4 Dataset Modern periodic words in the UK. Seasonally periodic words that appear in the modern UK corpus, but are not present periodically in the UK historical corpus.
(CSV)

S1 Table Strongly periodic words by category in the UK. Periodic words that occur in the UK historical corpora with at least 20% of their variance explained by a single component, grouped by topic category.
(PDF)

S2 Table Strongly periodic words by category in the US. Periodic words that occur in the US historical corpora with at least 20% of their variance explained by a single component, grouped by topic category.
(PDF)
