# Supplementary material for: Discovering Periodic Patterns in Historical News
Source: PLoS One. 2016 Nov 8;11(11):e0165736. doi: 10.1371/journal.pone.0165736 (PMC5100883; doi:10.1371/journal.pone.0165736)
Supplement: S1 Table — Periodic words that occur in the UK historical corpora with at least 20% of their variance explained by a single component, grouped by topic category. (PDF) [file pone.0165736.s005.pdf]

S1 Table. Strongly periodic words by category in the UK.

Category	Word Stems
Activities	accompanist, anniversari, archeri, audienc, band, boat, christma, concert, duet, encor, excurs, excursionist, exhibit, fete, firework, gala, journei, lectur, luncheon, marque, match, parad, park, pianofort, picnic, promenad, quartett, recit, regatta, seasid, shorn, soire, solo, song, steeplechas, swam, swim, tent, tourist, trip, violin, visit, visitor, walk, yacht, yard
Agriculture	crop, enclosur, ew, field, harvest, iamb, lamb, meadow, pastur, plant, ram, reap, reaper, ripen, shear, shearl, sheav, yield
Disease	asthma, bronchiti, cough, lozeng
Fashion	muff, overcoat
Flowers	muff, overcoat, begonia, bloom, bouquet, carnat, chrysanthemum, dahlia, fern, flower, foliag, fuchsia, geranium, greenhous, marigold, nurseri, orchard, pansi, rose
Food	appl, apricot, asparagu, cabbag, carrot, cherri, cucumb, currant, duckl, fruit, gooseberri, grape, lettuc, mackerel, marrow, melon, nectarin, onion, peach, pear, plum, raspberri, rhubarb, ripe, salad, soup, strawberri, tomato, trout, veget
Hunting	foxhound, gees, grous, harrier, hound, kennel, partridg, pheasant, rabbit, shoot, snipe
Politics	adjourn, amend, appoint, assiz, bill, claus, committe, debat, motion, propos, proposit, unanim
Sport	batsman, batsmen, bowl, bowler, cesarewitch, challeng, competit, competitor, cricket, croquet, dribbl, furlong, goal, handicap, haymak, ibw, jump, lbw, notout, out, pitch, polo, prize, race, round, run, runout, scorer, scrimmag, scull, sport, starter, stroke, stump, swimmer, tenni, try, wicket, winner, wkt, won
Time	afternoon, apnl, apr, apri, aprii, april, aug, augu, august, autumn, biennial, dec, decemb, easter, feb, februari, halfyear, inst, jan, januari, jau, jnly, jnne, juii, juli, julv, june, juno, juue, lst, mai, mar, march, monthli, nov, novemb, oct, octob, ootob, pril, quarterli, second, sept, septemb, spring, summer, tember, ult, whitsun, whitsuntid, winter, wintri, yearl
Weather	breez, cold, cool, foggi, frost, frosti, frozen, gale, heat, lightn, rain, shower, showeri, sleet, snow, sunshin, thunder, thunderstorm, weather
Other	2nd, 3rd, 9st, adopt, agre, almanac, almanack, appreci, aquat, ascent, aster, auditor, award, balanc, balmor, basket, best, bine, brace, brood, bunch, bye, caught, clip, collect, colt, common, cottag, covert, cronstadt, cut, deo, dialogu, discuss, distanc, dive, enjoy, entri, even, extra, ferret, filli, fine, first, five, foal, four, garden, geld, gimmer, glee, goodwood, grass, green, grils, ground, herbac, hors, horticultur, hous, humor, inspect, institut, introduc, lantern, lawn, leg, leger, length, leve, literari, lunch, mare, mayoralti, member, mile, move, mower, negativ, oppos, overs, parasol, peck, petit, picturesqu, plate, pod, poni, qualiti, quoit, rang, reynard, rune, schoolroom, scratch, singl, six, skeg, specimen, stake, start, supper, third, three, total, touch, verbena, welter, withdrawn
