# Supplementary material for: Discovering Periodic Patterns in Historical News
Source: PLoS One. 2016 Nov 8;11(11):e0165736. doi: 10.1371/journal.pone.0165736 (PMC5100883; doi:10.1371/journal.pone.0165736)
Supplement: S2 Table — Periodic words that occur in the US historical corpora with at least 20% of their variance explained by a single component, grouped by topic category. (PDF) [file pone.0165736.s006.pdf]

S2 Table. Strongly periodic words by category in the US.

Category	Word Stems
Activities	artist, banquet, beach, boat, circu, climb, comedi, comedian, cottag, dramat, drive, entertain, excurs, exhibitor, firework, hammock, holidai, masquerad, matine, opera, parad, parti, pavilion, picnic, regatta, seashor, seasid, sled, sleigh, surf, symphoni, tent, theater, theatr, visit, visitor, yacht, band, chautauqua, lectur, park, pianist, read, skate, soprano, swim, trip, vacat, violinist, barbecu, bather, cursion, excur, sonata, swimmer
Agriculture	crop, field, harvest, pastur, plant, ripe, seed, straw, thresh, weed, acreag, buckwheat, millet, reaper, thresher, wheat, scyth, sprinkler
Disease	bronchial, colic, cough, diarrhoea, dysenteri, throat, typhoid, croup, pneumonia, pneu, monia
Fashion	clearanc, cloak, cloth, coat, curtain, decor, fleec, latest, millineri, mink, muff, newest, plush, style, stylish, tailor, trim, velvet, waist, wear, woolen, cashmer, flannel, foulard, fur, gingham, merino, minc, mitten, overcoat, chambrai, chinchilla, ermin, furrier, muffler
Flowers	blossom, flower
Food	cucumb, egg, fish, melon, oyster, poultri, radish, sausag, soda, strawberri, appl, asparagu, beer, blackberri, cranberri, cream, lemonad, lettuc, pea, pumpkin, watermelon, cantaloup, gooseberri, grenadin, rhubarb, spinach
Hunting	beaver, gees, hog, quail, trout, game, rabbit
Politics	adjourn, bill, chairman, congression, legi, legisla, legislatur, resolut, debat, judiciari, legisl, lobbi, senat, dential, gression, presidenti
Sport	catch, catcher, fan, jump, out, pass, pitch, pitcher, stump, throw, winner, cricket, footbal, goal, race, saratoga, score, tenni, umpir, won, croquet, grounder, hockei
Time	aprll, august, autumn, birthdai, dec, decem, decemb, feb, februari, jan, jane, janu, januar, jnly, juii, jul, juli, jun, juno, mai, novemb, oct, oet, pec, ruari, sep, septem, septemb, spring, summer, sundai, tember, uari, ult, vember, winter, year, apr, april, aug, cember, deo, ept, fall, febru, januari, jau, julv, june, mar, march, mav, mber, nov, novem, ocl, octo, octob, sept, tober, apri, aprl, augu, auguat, bept, februar, feh, jali, jnne, juna, juue, jyl, keb, midsumm, oci, peb, pril, sepl, sommer, sopt, xov
Weather	blizzard, cool, drought, freez, heat, hot, lightn, sleet, snow, sun, rain, breez, cold, colder, cooler, frozen, gust, shad, shadi, thunder, coldest, coolest, hottest, inclement
Other	2b, 2s, 3b, ap, appropri, around, asburi, bath, beaten, bummer, bunt, chariti, christina, clip, committe, convent, crab, crate, crowd, dash, discuss, down, drill, drouth, drown, dusti, encamp, fab, fair, fare, fly, foal, fodder, fourth, gather, gauz, glen, graduat, ground, handicap, hit, hous, incorpor, indoor, inli, intro, joint, kersei, latur, lec, lution, matter, mitte, mosquito, mower, msy, nainsook, niagara, nic, night, nobbi, oc, off, orat, organdi, oxford, pace, parasol, pattern, percal, piqu, place, preparatori, present, propriat, prospect, pull, refer, refriger, reopen, resort, reunion, sabl, second, sen, snake, speed, sprinkl, squirrel, start, starter, stove, straight, stretch, stroke, supper, surround, swarm, synod, thei, trot, turn, wash, washabl, where, while, airi, almanac, apl, appro, appropria, ari, awn, base, batist, beat, ber, blanket, bug, buggi, bunch, calendar, candid, canva, caught, dee, duster, ember, fastest, garden, grass, heater, hors, hummer, imperson, insect, introduc, jel, lake, lawn, left, leghorn, lisl, member, messag, mountain, ocean, pend, pic, pick, platform, ponge, priation, provid, rake, recreat, reduc, refresh, relat, reso, resolu, return, ride, round, run, sheer, shower, stop, struck, suggest, sulphur, swing, thirsti, ticket, toi, troduc, water, zero, adirondack, baae, baso, carpet, debutant, decern, enow, freezer, goer, kraut, lenten, lynx, mareh, maroh, myl, navel, oversho, presbyteri, rattlesnak, sealskin, sunburn, thermomet
